# A minimally invasive fin scratching protocol for fast genotyping and early selection of zebrafish embryos

**DOI:** 10.1038/s41598-022-26822-7

**Published:** 2022-12-30

**Authors:** Martina Venditti, Catia Pedalino, Marion Rosello, Giulia Fasano, Malo Serafini, Céline Revenu, Filippo Del Bene, Marco Tartaglia, Antonella Lauri

**Affiliations:** 1 Genetics and Rare Diseases Research Division, Ospedale Pediatrico Bambino Gesù, IRCCS, 00146 Rome, Italy; 2 Sorbonne Université, INSERM, CNRS, Institut de la Vision, 17 Rue Moreau, 75012 Paris, France; 3 Institut Curie, PSL Research University, INSERM U934, CNRS UMR3215, 75005 Paris, France

**Keywords:** Biological techniques, Animal disease models, Animal breeding

## Abstract

Current genetic modification and phenotyping methods in teleost fish allow detailed investigation of vertebrate mechanisms of development, modeling of specific aspects of human diseases and efficient testing of drugs at an organ/organismal level in an unparalleled fast and large-scale mode. Fish-based experimental approaches have boosted the in vivo verification and implementation of scientific advances, offering the quality guaranteed by animal models that ultimately benefit human health, and are not yet fully replaceable by even the most sophisticated in vitro alternatives. Thanks to highly efficient and constantly advancing genetic engineering as well as non-invasive phenotyping methods, the small zebrafish is quickly becoming a popular alternative to large animals’ experimentation. This approach is commonly associated to invasive procedures and increased burden. Here, we present a rapid and minimally invasive method to obtain sufficient genomic material from single zebrafish embryos by simple and precise tail fin scratching that can be robustly used for at least two rounds of genotyping already from embryos within 48 h of development. The described protocol betters currently available methods (such as fin clipping), by minimizing the relative animal distress associated with biopsy at later or adult stages. It allows early selection of embryos with desired genotypes for strategizing culturing or genotype–phenotype correlation experiments, resulting in a net reduction of “surplus” animals used for mutant line generation.

## Introduction

The quick implementation of genomic sequencing in the clinical setting has allowed the identification of an unanticipatedly high number of candidate new disease genes, and variants implicated, for instance, in rare and previously undiagnosed pediatric diseases. The required fast and reliable methods to validate the functional significance of the findings and generate precise disease modeling needed for mechanism investigation and therapy innovation cannot bypass animals’ research. Indeed, the scientific solidity of an in vivo verification of genotype–phenotype association and the availability of whole organ/organismal disease models are not fully replaceable by in vitro approaches. Nevertheless, a cultural change towards rationalizing animal-based research and improving animal welfare conditions is being increasingly embraced at the national and international levels. Consequently, legal requirements for animal use in research have increasingly tightened in recent years^1–6^. Rightly, according to the 3R principle (“Reduce”, “Refine” and “Replace”)^3^, researchers should avoid uncritical use of animals in research. Instead, refined husbandry and strategic experimental protocols should be preferred to ensure animal welfare and reduce unnecessary burdens.

Alternative methods should also be considered when scientifically valid. In this sense, using small and transparent teleost fish embryos, larvae, and fry -when appropriate- represents a valid vertebrate alternative to large and invasive animal experimentation. Teleost fish have accompanied mice and frog models for more than three decades in discovering crucial vertebrate developmental processes. With their amenability to handling and culturing, the rapid and external development, the availability of efficient genetic manipulation techniques, and accessibility to whole embryogenesis in see-through strains, invasive procedures can be avoided in fish. Morphological and behavioral assessments relevant to human diseases can be performed in fish starting from embryonic stages, requiring minimal manipulation by the researcher and within a small experimental time window, minimizing animals’ distress. Notably, sophisticated mutant generation techniques^7–10^ and gentle imaging modalities, which do not necessitate surgery nor disturb animal physiology^11^, have advanced particularly for the most popular teleost model, zebrafish (*Danio rerio*). Together with the inter-laboratory harmonization of husbandry methods worldwide and the continuously updating welfare standards^12,13^, these features are driving the rising success of zebrafish in biomedical research. For instance, zebrafish embryos and larvae now serve as efficient animal alternatives as part of large functional genomics workflows to swiftly validate new disease genes and variants discovered in patients, generate advanced disease models^14,15^ and test novel therapeutic agents^16–18^.

The versatile application of genetic engineering innovations in zebrafish is at the center of this success, allowing to generating and improve disease models on demand. Transient or stable transgenic fish labeling specific cell populations and tissues or subcellular structures are readily and efficiently produced by transposon-mediated transgenesis and TALENs- or CRISPR/Cas-based gene knock-out or knock-in, nowadays routinely used to generate disease models^19–21^. In addition, gene editing techniques for targeted modifications are being established in this model^22–24^ and promise to generate precise disease models. Of note, an extensive collection of lines is available to researchers at certified international or European stock centers^6,25^. Moreover, annotations relative to all the available genetic and phenotypic information of a given disease model cross-referenced to OMIM are now implemented in popular zebrafish databases^26^.

In the context of animal welfare, the high efficiency reached by most of the genomic editor techniques in zebrafish permits substantial genomic modification already in the first-generation mutants, such that phenotypes in these G0 fish often recapitulate to a great extent those obtained by germline transmission in stable lines^27^. Mutant fish can thereby provide useful and preliminary information already within one round of initial experiments, minimizing the number of animals used for line generation and maintenance and/or analyzed at later stages. This advantage is of critical importance if considering the serious ethical concerns arising in connection with the culling of “surplus” animals needed solely for generating mutant lines and not included in research protocols^28–30^. In Europe, this number reached circa 14 million between 2015 and 2017^29,30^. While generation of stable lines remains necessary, preliminary investigation of the disease mechanisms in G0 zebrafish embryos or early larvae stages is rapidly increasing, for example, in the context of functional genomics studies^15,31^ and allows a first glimpse into the possible effect of genes’ loss of function. This preliminary approach translates into a net reduction of unnecessary rounds of breeding (including F2/F3) and, thereby, generation of “surplus” individuals^27,32–34^.

To further optimize mutant line generation and to allow strategical employment of all G0 fish (including those with undesired genotypes) with a resulting reduction in the total number of fish, the establishment of refined approaches for genotyping at early stages with virtually no animal distress are crucial. A method for tail fin clipping in 3 days-post-fertilization (dpf) fish was recently published^35^. Despite allowing earlier selection of animals compared to standard genotyping protocols in adults, this approach still entirely relies on tail cut to obtain bioptic material and is not useful for situations in which embryos earlier that 3 dpf might be useful. Early stages indeed can already be phenotypically informative, for instance with respect to locomotor response (i.e. touch evoked response)^36,37^.

Here we report on a refined and fast method for isolating sufficient genomic DNA material to genotype single zebrafish embryos as early as 2 dpf by simply scratching the tip of the tail fin. Compared to the existing methods, this procedure is minimally invasive to the fish, compatible with normal development and early in vivo analyses (such as behavior), and is readily performed using standard laboratory equipment and material. We compare the sensitivity of this protocol that we called “fin scratching” (FS) to standard whole-embryo (WE) and fin clipping (FC) methods and demonstrate the robustness of the procedure by verifying the successful amplification of two different transgenic fragments and three endogenous gene fragments that differ in size. In addition, we show its applicability for genotyping CRISPR/Cas9-based mutants in their first-generation (crispants) and from stable fish incross. For the latter, we show specifically the versatility of the FS method with respect to situations in which mutant genotyping outcome depends upon successful Sanger sequencing or amplification of fragments varying in size with or without downstream enzymatic digestion. The optimized fin scratching method for zebrafish embryos genotyping contributes to reducing and refining the use of animals for line generation and research.

## Materials and methods

### Fish maintenance

Zebrafish *AB* and *NHGRI*^38^ were obtained from EZRC (European Zebrafish Resource Center), *Tg(SAGFF73A:Gal4;UAS:Gtuba)*^39^ from NBRP Zebrafish (National BioResource Project of Japan for Zebrafsh, RIKEN) and the Kawakami laboratory, at the NIG (National Institute of Genetics)^40^. *Lakritz* mutants were previously described^41,42^. Fish were cultured following standard guidelines that ensure animal welfare^43^. Fish were housed in a water-circulating system (Tecniplast) under controlled conditions (light/dark 14:10, 28 °C, 350–400 μS, pH 6.8–7.2) and daily feeding with both dry and live food (freshly hatched nauplii of *Artemia salina*) was ensured. All zebrafish embryos were raised at 28 °C in freshly prepared embryos culturing medium (E3). All animal experiments were conducted on additional embryos before the free feeding stage (5 dpf) resulting from standard breeding and line generation in accordance with ARRIVE guidelines (https://arriveguidelines.org) and approved by the Italian Ministry of Health (541/2019-PR) and the committee on ethics of animal experimentation of Sorbonne Université in accordance with French and European Union animal welfare guidelines (APAFIS#21323-2019062416186982).

### Generation and phenotyping of crispant fish harboring the p.W273* change in *tyr* and generation of stable plekhh1 KO fish

To introduce a STOP codon in the *tyrosinase* (*tyr*) genomic sequence by base conversion (C to T) we used the crRNA oligo 5′-CTTCCAGGATGAGAACACAG-3′ targeting codon Trp273 within exon 1 following the protocol established by Rosello et al.^23^. Briefly, the synthetic gRNA was produced by combining 200 pmol of crRNA (Alt-R CRISPR-Cas9 crRNA, IDT, Integrated DNA Technologies) and Alt-R CRISPR-Cas9 tracrRNA and incubating the resulting mix at 95 °C for 5 min. *AncBE4max* mRNA was produced in vitro. To this aim, the coding sequence of the pCMV: *ancBE4max*^44^ was subcloned into pCSDest vector^45^ using Gateway cloning by first subcloning it into pDONR211 with BP clonase II enzyme mix (ThermoFisher, 11789020) and then transferring the expression cassette into pCSDest using LR clonase II enzyme mix (ThermoFisher, 11791020). The resulting plasmid was linearized with *KpnI*, and mRNA was produced using Sp6 in vitro transcription using mMESSAGE mMACHINE SP6 Transcription Kit (ThermoFisher, AM1340) following manufacturer’ instructions and with the addition of 1 μl of GTP. Approximately 1 nl of a mix containing 40 pmol/μL of sgRNA (crRNA + tracrRNA) and the 500 ng/μl mRNA encoding *ancBE4max* were injected into one-cell stage embryos. Fish were screened and phenotyped for occurrence of pigmentation defects upon 48 h post fertilization (hpf) following Rosello et al.^24^, assigning a different score (1 and 2) based on the reduction of pigments (severity) mainly visible in the retinal pigmental epithelium (RPE) of the eye. Refer to main text and figures for the details.

Stable *plekhh1* knocked out (KO) fish line was previously generated in Del Bene’s laboratory via standard CRISPR/Cas9 technique by injection of two sgRNAs (formed starting from the crRNA oligos 5′-GCATATGAAACTCCCGGTCC-3′ and 5′-ATCGTGCCACAGTCTCGTCC-3′, Alt-R^®^ CRISPR-Cas9 crRNA, IDT, Integrated DNA Technologies) together with Cas9 protein at 15 µM (IDT).

### Capillaries preparation

**Figure 1:**
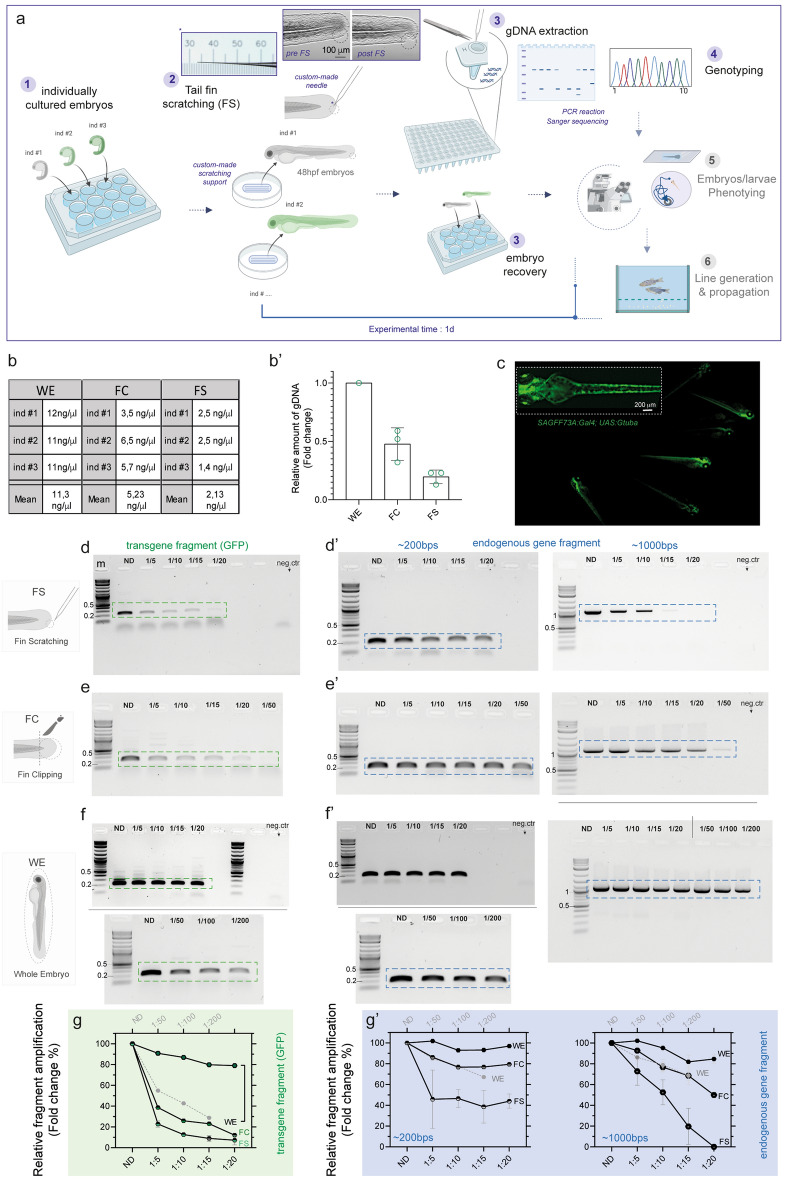
Caudal fin scratching protocol in 48 hpf zebrafish embryos allows sufficient genomic DNA extraction and genotyping. (**a**) Schematics of the general workflow to obtain genomic DNA (gDNA) from the tail fin scratching (FS) of individually cultured zebrafish embryos at 48 hpf. The single embryo are cultured in separated well plates (1); gentle tail fin scratching and tissue collection is performed on anesthetized embryos by using custom-pooled glass capillaries and a custom-made agarose-based support where fish are positioned (2); the tip of the capillary containing the collected tissue material is recovered and the samples are immediately processed to extract crude gDNA, while fish are recovered after the procedure (3); genotyping analyses are performed on the gDNA (4), while recovered embryos can immediately be used for morpho-functional phenotyping (5) and/or fish line generation and propagation (6). In step 2, a close-up of a representative custom-pooled needle with a reference scale. Insets on the right show representative bright field images of a single embryo before (pre-FS) and after (post-FS) the completion of the FS procedure. The extend of tissue removal after the procedure is shown in the dashed blue circle in post FS. (**b, b′**) Quantitative comparison of the yield of FS-, FC- and WE-derived gDNA from single embryos. Raw values from three different embryos (ind #) are shown in the table in b, the data are plotted as fold change relative to gDNA amount collected from WE-derived method in b’. The mean ± SEM from three individual embryos is shown. (**c**) Representative fluorescent image showing *Tg*(*SAGFF73A:Gal4;UAS:Gtuba)* embryos at 48 hpf, where green fluorescent protein (GFP) marks the ubiquitous expression of tubulin protein. A higher magnification on one of the embryos is shown in the inset on the top left. (**d, d′**) PCR results of fragment amplification of target GFP (**d**) and of endogenous gene fragment (**d′**) of approximately 200 bps (left) and 1000 bps (right) in length using non diluted (ND) or a series of dilutions of the original FS-derived gDNA from a single 48hpf embryo. (**e–f′**) PCR results of the same targets as in (**d**, **d′**) obtained by FC (**e**, **e′**) or by WE (**f**, **f′**) method from one single experiment. In each experiment a negative control (neg ctr) was added. One negative control was run for the PCR belonging to the same experiment (**e** and **f** or **d′**–**f′** for 200 bps). Different gels in (**f**) are demarcated by a black line. For each procedure (FS, FC and WE), a comparative schematic is shown on the left. For FS, a representative PCR result is shown from three (**d**) and two (**d′**, left and right) independent experiments. For FC and WE, the single PCR performed is shown in (**e**, **e′**) left panel and (**f**) and (**f′**) left panel (200 bps band). A representative result from two independent experiments is shown in e’ and f’ right panel (1000 bps band). (**g–g′**) Plot showing the quantification and the relative amount of fragment amplification for GFP (**g**) and endogenous control gene fragments (**g′**) resulting from PCR reactions using different dilutions of the FS-, FC- and WE- derived single embryo gDNA. Values representing the relative quantification of the target amplicons from different dilutions derive from densitometric analysis of the PCR bands visible on agarose gel and are plotted as percentage changes compared to the WE-derived not diluted sample. FS data are expressed as mean ± SEM of the replicates as indicated above. m = molecular marker (Quick-Load^®^ 1 kb Plus DNA Ladder, NEB #N0469S), the bands of 0.2, 0.5 and 1 kb are indicated. Source data are provided as a Source Data file.

Capillaries with a thin tip (Fig. 1a) were prepared following our standard laboratory protocol to generate microinjection needles for zebrafish embryos. Briefly, needles were custom pooled using commercial capillaries (1.0 OD × 0.58 ID × 100 L mm, 30-0019 CAPILLARIES GC100F-10, HARVARD apparatus) at the PC-100 NARISHIGE using a 2-step pooling protocol. The heating value representing the proportion (%) to the maximum output was set to 80 in step 1 and reduced to 75 in step 2. The pulling was achieved using a single heavy weight and the slider for the first pull set to 4 mm. Note that the conditions might vary depending upon the laboratory specific air and humidity conditions.

### Tail fin clipping and fin scratching procedures

The experimental workflow is illustrated in Fig. 1a and in Supplementary Fig. 1. Embryo clutches were collected after birth and each single embryo was washed several times in fresh E3 medium. Embryos were raised individually in separated wells and the E3 medium was changed every day before tail scratch to avoid cross-contamination of tissue between embryos. At the pharyngula stage (44–48 hpf), embryos were anesthetized with 0.1 mg/ml MS-222 (Pharmaq^®^ Ltd, 404841). Using a plastic Pasteur pipette with the tip cut-off embryos were gently transferred on a custom-made scratching support plate prepared with 2% agarose (Biochemical, #PCO701) in E3 and a pre-set mold (World precision instruments), following standard methods to generate microinjection plates^46^. A single fish was positioned within one groove and different grooves or different support plates were used for each fish to minimize cross-contamination of biological material. Excess surrounding E3 medium with MS-222 was removed before the procedure. For the tail (caudal) fin clipping (FC) protocol, each embryo was arranged laterally within the lane. A tiny portion of the caudal fin was cut using standard scalpel blade size 11 (PARAMOUNT surgical blades, C10344). For the caudal fin scratching (FS) protocol, each embryo was positioned similarly, and the end of the tail was gently scratched. To this aim, we used the sharp end of a glass capillary which was custom pulled (following the protocol to generate standard injection needles) and gently opened using forceps. The FS was obtained by using such pooled capillary and applying light pressure to scrape a little tissue portion from the fin, which resulted in a very small fin biopsy (Fig. 1a and Supplementary Fig. 1). We could not establish how much material was collected within the capillary by capillarity and how much remained instead attached to its outer walls. Therefore, the tissue material collection was ensured by breaking and recovering the final portion of the capillary into a sterile collection tube containing 100 μl of PBS 1× (Fig. 1a and Supplementary Fig. 1). Samples were stored at − 80 °C or processed immediately. After each procedure, the fish was quickly placed in fresh E3 medium for full recovery and subsequent use. Control genomic material from whole embryos (WE) was obtained by processing the entire embryo tissue at pharyngula stage.

### Genomic extraction, DNA amplification protocol and SANGER sequencing

Genomic DNA extraction was performed using the Genomic DNA Extraction & Purification kit (NEB #T3010) according to the manufacturer's Instructions. Briefly, for tissue disruption and cell lysis, the collected material in 100 μl of PBS 1 × was treated with 3 μl of RNAse A and 1 μl of Proteinase K provided by the kit and briefly mixed by vortexing and incubated in standard 100 μl of Lysis buffer for 5 min at 56 °C with agitation at 1400 rpm. After this treatment, genomic DNA was obtained by the manufacturer column-based binding and eluted in 30–50 μl of elution buffer. PCR for single embryo genotyping were performed using AccuPrime (ThermoFisher, #12,346,086) or Q5 high fidelity polymerase (NEB # M0491S). For DNA extractions resulting from WE, FC or FS preparations a total of 3–5, 8–10 and 15–20 μl (and different dilutions) of the extracted genome was used in each PCR reaction, respectively, for a total volume of 25 μl. 10 μl of the PCR product was analyzed on a 1.5% agarose/TBE gel. Primers used to amplify the different gene fragments are listed in Supplementary Table 1. The following touch-down cycles were used for PCR amplification: 73–69 °C for 30′′, 35 cycles in total (GFP fragment of approximately 230 bps); 66–63 °C for 30′′, 35 cycles in total (GAL4 fragment of 200 bp); 66–62 °C for 30′′, 35 cycles in total (first endogenous gene fragment of approximately 200 bps); 67–63 °C for 30’’, 40 cycles in total (second endogenous gene fragment of approx.530 bps); 70–66 °C for 45′′, 40 cycles in total (third endogenous gene fragment of approx.1000 bp); 70–66 °C for 30′′, 40 cycles in total (screening of G0 harboring base conversion in the genomic locus of *tyrosinase*); 71–67 °C for 30′′, 40 cycles in total (selection of heterozygous *plekhh1*^+/−^). In every PCR reaction the initial denaturation was performed at 98 °C for 1′, the denaturation for each cycle was set at 98 °C for 10′′, extension in each cycle was performed at 72 °C for 30′′ and the final extension was set at 72 °C for 2′. For the 2nd round of PCR on the PCR amplifying the 1100 bps endogenous fragment the initial genomic denaturation was performed at 98 °C for 1′ (first trial) and for 40′′ (second trial).

For Tyr (p.W273*), the resulting band of approximately 450 bps was purified by gel extraction and sequenced using Sanger sequencing using a 3500 Genetic Analyzer (Applied Biosystems). Sequences and chromatograms were assessed using Snapgene 5.3, Geneious and EditR^47^. For the screening of *plekhh1*^+/-^ the presence of a single 493 bp band indicated a wild-type embryo, and the co-occurrence of a 493 bp and a 200 bp band indicated a heterozygosity condition for the introduced deletion. For the screening of the *lakritz (lak)*^+/−^ mutant line^41,42^ after the entire 25 µl of the resulting PCR product was digested using 5 units of Stu I (NEB, R0187S) overnight, the presence of a 200 bps and 100 bps denoted a normal genotype (WT) while the presence of an additional 300 bps band indicated heterozygosity.

### Bright field, fluorescent image acquisition and data analysis

Bright-field and fluorescent images of live fish were acquired at Leica M205FA or Thunder Imager (Leica Microsystem), the latter with a PLAN 5X/0.12 PHO objective. Densitometry analysis of the bands corresponding to the relative amount of target amplification was obtained using Fiji tool^48^ on raw 8-bit agarose gel images. Data analysis and statistical assessments were performed using GraphPad Prism 9.4.1 s (GraphPad Software, San Diego, California USA, www.graphpad.com). Log-rank (Mantel-Cox) test was used to assess survival in zebrafish *plekhh1* mutants. Two-sided Chi-square’s test in 2 × 2 contingency table (wild-type *vs.* altered genotypes) was used to compare statistically the genotyping success from WE and FS preparations fromcrispant fish. Figures were assembled using Illustrator (Adobe) or Power Point (Microsoft Office 365).

## Results

### Caudal FS method allows extraction of genomic DNA from 2 dpf zebrafish embryos

We aimed to improve currently available protocols for genotyping zebrafish larvae^35,49,50^ with the goal of obtaining successful genotyping results by a) testing young individuals (embryos) and b) gentle procedures, such to “reduce” the number of free-feeding animals used for breeding purposes and to “refine” embryos selection for line generation and/or direct genotype–phenotype studies. Thereby we asked whether sufficient genomic material could be retrieved from the caudal fin already during embryonic stages and whether we could avoid the employment of invasive surgery (cutting or clipping) as much as possible. To minimize possible interference with developmental and morphogenesis processes, we chose embryos at the pharyngula stage (between 44 and 48 hpf), in which the dynamic segmentation period is nearly completed, trunk lengthening is slowed down, but a clear morphology of the caudal fin fold with typical collagenous fibers is apparent and can be visualized using standard bright-field or Nomarski microscopy^51^ (Fig. 1a and Supplementary Fig. 1). We reasoned that a minimal portion of caudal tissue might be removed by a gentle scratching using thin custom made glass capillaries—which we normally employ for microinjection—and simultaneously collected by retrieving the FS capillary and likely taking advantage of their capillarity effect. Fish at the pharyngula stage could be slightly anesthetized and placed on custom-made holders produced with 2% agar dissolved in E3 medium. Custom-pooled glass capillaries whose tip was open by forceps and measured between 20 and 30 µm (outer) and 9 µm (inner) diameter were applied gently to the tip of the ventral fin epithelium at the end of the tail to scratch and recover embryonic tissue material. The slightly scratched fin could be observed upon completion of the procedure (Fig. 1a and Supplementary Fig. 1), which usually lasted less than three minutes per embryo, from lining the embryo to obtaining the FS-derived tissue material and rescue of the embryo. The small bioptic material was collected by retrieving the tip of the capillary into a standard tube (Fig. 1a and Supplementary Fig. 1). Thereby, the whole FS procedure, genotyping and analysis for a sample of approximately 60 embryos can be performed within one experimental day. To assess whether the FS protocol was compatible with genotyping purposes, we extracted the genomic DNA from the collected material and we first compared it to the amount obtained using classical fin clipping (FC) protocol or whole-embryo (WE) genomic extraction. For all the conditions we used the same genomic extraction protocol employing standard commercially available-column-based gDNA purification. Quantification of the purified genomic material indicated that the DNA obtained from FS was approximately 2 ng/µl for a total of 60 ng (Fig. 1b), which on average is approximately 2- or 5- fold lower than that obtained with FC or WE preparations, respectively (Fig. 1b,b′).

### Validation of the efficiency and flexibility of genotyping via FS method

Next, we tested if the quality and quantity of the genomic material extracted by the caudal FS method are suitable for various genotyping needs and for multiple PCR reactions. We first explored the method's performance using transgenic *Tg(SAGFF73A:Gal4;UAS:Gtuba)* pharyngula embryos, which express GFP-tagged tubulin in the majority of the cells and tissues^52^ under the control of the Gal4-UAS system^39^ as positive control. We aimed at amplifying a small fragment (of approximately 200–300 bps in length) using specific primers targeting the GFP marker inserted into the genome of the established transgenic fish from FS-derived gDNA of single GFP positive embryos (Fig. 1c). While selecting for GFP in the double transgenic line can be readily achieved under the microscope and therefore it represents only a positive control for our PCR-based genotyping experiment; selecting fish expressing the non-fluorescent Gal4 transgene can only be done via PCR-based genotyping, and it is routinely performed in facilities for breeding purposes. Therefore, we also decided to test the amplification of Gal4 in the GFP negative embryos. We further tested the performance of our FS-based genotyping and set out to amplify fragments of increasing sizes (approximately 200, 500 and 1000 bps) of an endogenous locus investigated in the laboratory. To assess the performance of FS with respect to standard methods, we directly compared the PCR results obtained with different dilutions of the FS-derived genomic material with that obtained from classical FC and WE methods.

Using between 30 to 50 ng of genomic material in the PCR reaction and specific primers we were able to amplify the GFP fragment of the GFP + embryos (Fig. 1d), an endogenous fragment of approximately 200 bps (Fig. 1d′, left, Supplementary Fig. 2a), 500 and 1000 bps (Supplementary Fig. 2a and Fig. 1d′, right). From the gDNA extracted with the FS method, we obtained detectable fragment amplification even using dilutions of the original genomic material for all the different sizes (Fig. 1d,d′). As anticipated by the quantification of the gDNA (Fig. 1b,b′), a higher accumulation of amplicons was obtained by using FC and WE-derived gDNA as starting material (Fig. 1e–g’), which gave rise to detectable amplification even with higher dilution factors (Fig. 1g,g′). As expected, however, we observed a decrease in the amplification success with larger gene fragments (Supplementary Fig. 2a and b), which also performed worst in the highly diluted samples (both for FS and FC methods) (Fig. 1d′,e′, right). In addition, we also observed amplification of Gal4 fragment in a fraction (65%) of GFP negative fish from the *SAGFF73A:Gal4* × *UAS:Gtuba* incross, in which we could amplify the control endogenous 200 bps fragment (Supplementary Fig. 3a), which denotes successful genomic extraction.

These results indicate that, despite the limited amount obtained compared to standard FC- and WE-based tissue collection, the genomic material derived from the FS protocol in zebrafish embryos is sufficient for different rounds of genotyping using a dilution of the original preparation, for instance, to assess different genetic modifications. The data also confirm that, likewise other genotyping methods, the amplification of small test fragments (< 1000 bps) is preferable.

Next, with the aim to assess whether resampling from the same individual might be avoided when using FS, we tested if multiple rounds of PCR could be used to expand the genotyping possibilities or re-test negative results for control fragments (endogenous loci) likely due to the scarce and variable (and/or of potentially low quality) amount of gDNA and transgenic (*i.e.* Gal4). The “PCR on PCR” experiment confirmed efficient and specific re-amplification of the small gene fragments, also when the first round gave negative results (Supplementary Fig. 4a,b, and Supplementary Fig. 3b). For larger fragments the “PCR on PCR” did not yield particularly successful results in the first trial, which showed a smear signal, likely due to the poor performance in amplifying large fragments. However, a slight but useful improvement could be obtained by varying the “PCR on PCR” cycling parameters (Supplementary Fig. 3c).

Altogether, these data show that FS-based genotyping is in principle compatible with multiple rounds of amplification on residual amplicons from the first PCR reaction, and that case-specific improvements of the PCR conditions might be required. Genotype information can be therefore recovered from residual low-quantity genomic DNA samples, without the need to resample from the same fish or use more individuals, in full agreement with the 3R principle. This “two-round PCR approach” can also be used for preparative purposes aimed at amplifying the target sequence for follow-up experiments, permitting for instance sequencing and cloning.

### Efficient genotype selection of stable and mosaic G0 mutant embryos using FS method

**Figure 2:**
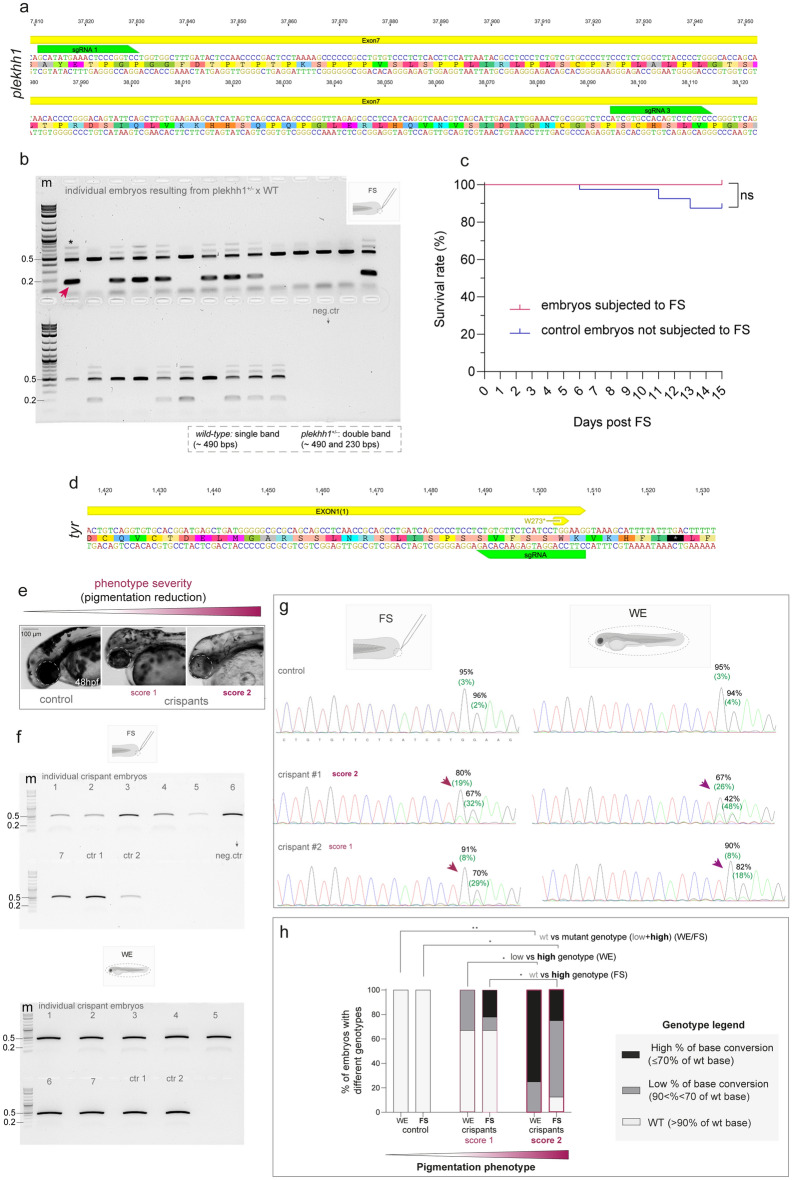
Efficient genotyping of 48 hpf transient and stable CRISPR-Cas mutants using genomic DNA derived from the FS protocol (**a**) Schematics of the zebrafish genomic sequence showing the position of guide-RNAs used to generate the *plekhh1* mutant line. (**b**) PCR result showing the possibility of genotype segregation using the FS-derived gDNA of single embryos from the offspring of *plekhh1* mutants outcrossed with WT fish. A fragment of the *plekhh1* exon targeted by CRISPR-Cas is amplified. Wild-type (single band, ~ 493 bps) are discernable from heterozygous *plekhh1*^+*/-*^ mutants (double band, ~ 493 and 230 bps, red arrowhead). The asterisk shows not specific bands. The agarose gel shows the result of a single PCR experiment. (**c**) Survival rate of embryos subjected to FS compared to control individuals from the same clutch, which were not subjected to FS. Log-rank (Mantel Cox) test is used to assess the statistical significance between groups (ns = not significant). Data show the survival rate from a single experiment with n = 40 control embryos non subjected to FS and 24 embryos subjected to FS. (**d**) Schematics of the zebrafish genomic sequence showing the position of the guide-RNA used to generate the *tyr* crispant with the base conversion (C > T, here G > A on the complement strand) leading to W273*. (**e**) Representative phenotypes showing partially (score 1) or severely (score 2) depigmented crispant mutants at 48hpf obtained upon *tyr* base editing. Reduced melanin content is apparent compared to a not-injected control sibling shown on the left. The images are representative of embryos from a single batch. (**f**) PCR results showing successful amplification on different crispants of a target fragment surrounding the edited *tyr* exon using the FS-derived gDNA (upper panel). Amplification of the same target sequence obtained by the gDNA extracted from the remaining WE tissue of the same individuals is shown in the lower panel. The agarose gel shows the result of a single PCR experiment. For both FS and WE two not-injected controls (ctr1 and ctr2) are also shown. (**g**) Examples of chromatograms resulting from Sanger sequencing of amplicons from WE- and FS- derived gDNA of the same *tyr* base edited crispants at 48 hpf. Examples of the performance of FS compared to WE for score 1 and score 2 are shown. Visible base conversion (here G > A) leading to a premature STOP codon is indicated by a purple arrow. Percentage of conversion G > A (C > T) and of residual G were calculated by EditR and are shown in green and black, respectively. A representative WT chromatogram from a non-injected sibling is also show on the top as negative control. m = molecular marker (Quick-Load^®^ 1 kb Plus DNA Ladder, NEB #N0469S), the bands of 0.2 and 0.5 kb are indicated. (**h**) The bar graph shows the genotype–phenotype correlation obtained by WE or FS-derived genotyping. Incidence (%) of sequences from *tyr* crispant embryos of different severity obtained by WE or FS exhibiting altered genotype (low or high rate of base conversion) compared to WT controls. N = 2 (controls), 9 for score 1 and 8 for score 2. Two-sided Chi-square’s test in 2 × 2 contingency table (WT *vs.* altered genotypes) are used to assess statistical significance between the different groups. Adjusted P values are reported (*p < 0.05, **p < 0.01). Source data are provided as a Source Data file.

Next, we set out to test the utility of the technique also for sorting mutant fish already during embryonic stages.The selection of individual animals carrying the desired genetic modification is a necessary step in generating and propagating mutant lines, whether from the microinjected G0 embryos or from the offspring originating from stable mutant F1–F3 adult fish crosses, with the final aim to establish informative genetic models. Genotyping is thus essential to isolate specific genotypes (*e.g.* heterozygous or homozygous) such to perform genotype–phenotype correlations and for strategizing as early as possible the use of mutant and “surplus” individuals in experiments. We, therefore, assessed the performance of FS-based genotyping obtained from 48 hpf progeny of stable mutant lines outcrossed with wild-type AB fish. For example, in one line carrying a KO allele for plekhh1*,* a protein involved in actin cytoskeleton control^53^ and recently generated in the laboratory using CRISPR-Cas9, genotyping is determined by the different sizes of wild-type and mutant amplicons (Fig. 2a). We also tested the performance of the FS method to screen an additional stable line^41,42^ (*lak*, carrying a KO allele for *atho5*) for which the genotype is instead determined by fragments analysis upon enzymatic digestion of PCR product (Supplementary Fig. 5).

We could successfully genotype all tested embryos (n = 24 for *plekhh* and n = 10 for *lak*) on which tail FS and genomic extraction were performed and isolate heterozygous animals (Fig. 2b and Supplementary Fig. 5) for future experimental needs and/or mutant line maintenance. The FS protocol applied to *lak* mutants shows that the method is also compatible with genotyping requiring downstream enzymatic digestion of the PCR product. Of note, by applying early FS, only 33% and 40% of the total embryos (for *plekhh1*^+*/−*^ and *lak*^+*/−*^, respectively) would be raised until adulthood to the aim of line propagation. We also confirmed the compatibility of the procedure with embryos development. Indeed, survival of fish, which was assessed until juvenile stages, was not affected in fish from the *plekhh1* mutant screening subjected to FS compared to siblings not involved in the procedure (Fig. 2c).

The genomic material extracted by our FS represents only a few cells of the tail tissue. Therefore, we next investigated if such scarce and variable cell representation could practically assist selection of 48 hpf crispant embryos, which, contrary to stable mutants, have only a limited and variable number of cells within the body harboring the desired indel(s)/mutation(s). In addition, given the frequent necessity of sequence information, we were also interested in testing whether Sanger sequencing could be compatible as a downstream reaction with the quality of the amplicons derived from our FS method. To this aim, we performed FS-based genotyping on G0 obtained using CRISPR-Cas9 DNA base-editing (Be) technology^54^ that produces C:G to T:A conversions into the genome^55,56^ recently implemented in zebrafish by Del Bene’s team to introduce a premature STOP codon in the *tyrosinase* (*tyr*, W273*) (Fig. 2d). The resulting reduced function of the tyrosinase enzyme generate partially depigmented mutants in G0, which are easily selected under the microscope (Fig. 2e) and served as positive crispant controls whose mutation could also be detected via Sanger sequencing^23,24^ (Fig. 2d–h).

Given the high probability of sampling non-mutant cells from the tail FS method in the mosaic G0 embryos, we used WE-based preparations as internal controls, while also asking how FS performance compares to a standard WE-based genotyping in representing the variable efficiency of genomic modification normally observed in a G0 mosaic population. First, as expected, we obtained fish with variable degrees of pigmentation left that we scored as showing a score “1” or “2” based on phenotype severity clearly observed in the RPE of the eye (Fig. 2d and Supplementary Fig. 6). The FS preparations allowed sufficient amplification of the target *tyr* locus (Fig. 2f) and successful and accurate target sequencing (Fig. 2g and Supplementary Fig. 6) for the majority of the samples (19/22 compared to 20/22 for WE). In terms of genotype–phenotype correlation performance, as expected, we observed a good match from the sequencing results of WE preparations (Fig. 2h). Despite to a lesser degree, the occurrence of the genomic modification in the chromatograms could also conveniently be assessed from FS-derived genotyping of the same individuals. Indeed, the occurrence of altered sequences (“high” + “low” conversion levels altogether) was not significantly different from WE (Chi-square test between FS and WE, Fig. 2h). Noteworthily, when considering the overall observed base conversion efficiency, we could distinguish *score 1* and *score 2* phenotypes both from WE and FS preparations (Fig. 2h), indicating a certain degree of genotype–phenotype correlation. However, as expected, compared to WE-preparations, FS-derived gDNA failed to assign “high” *vs.* “low” rate of base conversion in the *score 2* and *score 1* phenotypic classes, respectively (Fig. 2h). Contrary, for instance, in the FS preparations one fish labaled as *“score 1”* showed “high” base conversion levels, indicating enrichment of enginnered cells within the tail region. In comparison, the majority of fish labaled as *“score 2”* showed a relatively “low” base conversion level. In our specific example, the latter is expected when sampling a small population of cells from the tail tip in a mosaic fish which was scored based on the most visible pigmentation phenotype in the head.

Altogether, these results demonstrate the flexibility and limitations of our tail FS method to obtain enough good quality genomic material for both stable and mosaic mutant selection already during embryonic stages.

## Discussion

The scientific community is currently witnessing increasing attention toward animal protection and welfare practices, which is quickly being translated into incrementally stringent legal requirements for using animals in research. This ethical awakening calls for developing advanced procedures and methodological strategies that minimize the number—and concurrently optimize the use—of animals involved in experimental protocols or bred to establish mutant lines (“surplus animals”). Importantly, the generation and the destiny of the latter start to be recognized as a significant ethical concern that we must deal with^28,29^.

Even if promising in vitro and ex vivo methods that could serve as alternative approaches to animals are being proposed^57,58^, abolishing in vivo models entirely without affecting the quality and solidity of the scientific tests and findings remains inconceivable. For instance, verification of the causal link between putative pathogenic variants and disease often requires organismal-based functional validation, mainly when dealing with a gene for which the function of the encoded protein is not fully characterized. In addition, animal-based disease modeling is needed for preclinical research to assess new drugs for therapeutic use.

The freshwater fish zebrafish and other small vertebrate models have the potential to better the conditions of animal use in research towards improved ethical standards. Their amenability to minimally invasive or non-invasive procedures and their exploitability in terms of efficient disease model generation and phenotyping can be explored further to reduce (or better strategize) the use of large animals or the burden associated with invasive techniques typically required in adult fish.

With these needs in mind, this study aimed at establishing a rapid technique to obtain genomic material of good quality and sufficient quantity from zebrafish embryos for various genotyping purposes by using the least invasive in vivo procedure and permitting individual fish selection at early stages (during embryogenesis) with minimal animal distress and physiological alterations. We describe all the steps of a simple protocol of fin scratching (FS) that we designed to obtain a limited tissue scrape from the tail of single embryos as early as 2 dpf by soft tissue scratch and simultaneous collection using a custom-pooled glass capillary.

We demonstrate that the gDNA extracted from the FS-derived tissue at this stage is sufficient for various genotyping needs. Our protocol differs fundamentally from the standard fin clipping (FC) routine classically used for fish genotyping and more recently proposed methods^59^. It represents an improvement concerning different aspects of animal welfare in line with the 3R principle^60^. Conversely to the method presented here, in the consolidated FC technique, typically carried out in young adults as part of a breeding protocol, a considerable portion of the caudal fin tip is removed by a scalpel cut. Of note, Kosuta et al. (2018)^35^ recently showed that FC-derived genotyping could be successfully performed already in hatching larvae at 3 dpf and, therefore, before the legally protected free-feeding stage. Nevertheless, these young larvae are still subjected to a relatively invasive procedure on the tail that does not allow early functional assessment, for instance, of informative behaviors, which, as discussed, can occur as early as 2dpf^36,37^. Zhang et al.^59^, have proposed a method to obtain sufficient gDNA directly from skin cells of live zebrafish by enzymatic treatment of the entire fish. The resulting genotyping is limited with respect to the sampled cell population. Moreover, given the treatment of live embryos with proteases, it is still likely to produce considerable distress and possibly impact animal physiology. Instead, we demonstrate that a gentler, minimally invasive and fast procedure (representing an improvement according to the “Refinement” principle^2^) can be successfully carried out in earlier stages. Our FS protocol minimizes distress and possible physiological alterations, anticipates the selection of desired individuals, and therefore can be applied to rationalize (and reduce) early-on animal use for follow-up experiments or mutant line generation. The experiments performed here also showcase the robustness, gentleness, flexibility and limitations of the developed procedure.

The data presented demonstrate that, despite the small yield of gDNA obtained by FS embryos compared to standard approaches, it can support specific target amplification of single 2dpf embryos with respect to simple or more complex endogenous and transgenic gene fragments, as well as multiple rounds of genotyping. Indeed, dilutions of the original gDNA can be successfully employed in different PCR reactions, and additional rounds of amplifications starting from the first PCR (“PCR on PCR”) can be performed. This possibility altogether reduces the need for resampling and the potential distress it causes to developing animals. Importantly, our data also suggest that amplification of small fragments should be preferred to ensure a reasonable success rate when screening multiple fish, similarly to standard genotyping methods such as FC. We also prove the compatibility and limitations of the FS-derived gDNA with respect to accurate genotype segregation in stable lines with no impact on animal development/ survival and in transient mutants exhibiting a mosaic pattern of genomic modification. It should be noted that the early genotyping and selection of embryos with the desired genomic modification guaranteed by our FS protocol is ideal for reducing the husbandry and growth of "surplus" animals and for rationalizing their use already from embryonic stages. If selected early on, animals without the desired modification can be promptly rerouted for other experimental needs, without constituting an ethically unacceptable waste^28,29^.

As mentioned, increased experimental employment of the first generation of genetically modified zebrafish (crispants, G0) at embryonic and larval stages is conveniently to collect preliminary information on the gene loss of function^61^. It contributes to the further reduction (and smart planning) of the total number of animals needed for mutant line generation and analysis. We show that our FS procedure for obtaining gDNA is compatible with Sanger sequencing reaction and demonstrate its usefulness in accurately detecting G0 fish carrying the desired genetic modification (a base conversion in the case shown here). Of note, when we enquired more closely the genotyping-phenotyping correlation performance obtained by assessing the small tail tissue recovered by FS we could practically distinguish *score 1* and *score 2* fish. However, compared to WE preparations, the performance in resolving fish with “high” versus “low” levels of base conversion overall was poor. This is expected when employing limited embryo material, given that the phenotype pigmentation severity of the embryos was assigned based on the retinal pigmented epithelium, which is not represented in the tail tissue. We expect a similar result from any procedure focused on collecting a small biopsy, including standard methods such as FC. Nevertheless, the data demonstrate that the FS method established in this study can support gentle and fast embryo selection of crispant fish with a limited capability to differentiate phenotypes ‘severity. This minimally invasive genotyping technique in early embryos might be, therefore, a valuable tool to select and characterize preliminarily early-on severe mutants, which might suffer significant discomfort during a FC procedure and whose phenotype might worsens with growth, reducing the possibilities of investigation.

In conclusion, the FS protocol established here allows fast, cheap and accurate genotyping from zebrafish embryos already at 2 dpf, readily implementable in any laboratory setting.

Improving currently used methods and permitting genotype segregation during embryogenesis, this procedure contributes to reduce and refine the use of animals in research.

## Supplementary Information

Supplementary Information 1.

Supplementary Information 2.

## Data Availability

Uncropped raw gel images and the datasets generated during the current study are provided in the Source data file and in Supplementary material. The raw Sanger sequencing chromatograms are available in the Dryad database (https://doi.org/10.5061/dryad.qv9s4mwjc). Raw bright-field images are available from the corresponding author upon request.

## References

[1] Aleström P (2020). Zebrafish: Housing and husbandry recommendations. Lab. Anim..

[2] Hubrecht RC, Carter E (2019). The 3Rs and humane experimental technique: Implementing change. Animal (Basel).

[3] Russell WMS, Burch RL (1959). The Principles of Humane Experimental Technique.

[4] Guillen J (2012). FELASA guidelines and recommendations. J. Am. Assoc. Lab. Anim. Sci..

[5] European Parliament and the Council of the European Union. Directive 2010/63/EU of the European Parliament and of the Council of 22 September 2010 on the protection of animals used for scientific purposes. Official Journal of the European Union L 276. **53**, 33–79 (2010).

[6] Geisler R, Borel N, Ferg M, Maier JV, Strähle U (2016). Maintenance of zebrafish lines at the European zebrafish resource center. Zebrafish.

[7] Satou C (2022). A viral toolbox for conditional and transneuronal gene expression in zebrafish. Elife.

[8] Watakabe I (2018). Highly efficient generation of knock-in transgenic medaka by CRISPR/Cas9-mediated genome engineering. Zool. Lett..

[9] Sassen WA, Köster RW (2015). A molecular toolbox for genetic manipulation of zebrafish. AGG.

[10] Liang JO, Kornfeld S (1997). Comparative activity of ADP-ribosylation factor family members in the early steps of coated vesicle formation on rat liver Golgi membranes. J. Biol. Chem..

[11] Abu-Siniyeh A, Al-Zyoud W (2020). Highlights on selected microscopy techniques to study zebrafish developmental biology. Lab. Anim. Res..

[12] Goodwin N (2016). Standardized welfare terms for the zebrafish community. Zebrafish.

[13] Lee CJ, Paull GC, Tyler CR (2022). Improving zebrafish laboratory welfare and scientific research through understanding their natural history. Biol. Rev. Camb. Philos. Soc..

[14] Siekierska A (2019). Biallelic VARS variants cause developmental encephalopathy with microcephaly that is recapitulated in vars knockout zebrafish. Nat. Commun..

[15] Bonora E (2021). Biallelic variants in LIG3 cause a novel mitochondrial neurogastrointestinal encephalomyopathy. Brain.

[16] Ganzen L (2021). Drug screening with zebrafish visual behavior identifies carvedilol as a potential treatment for an autosomal dominant form of retinitis pigmentosa. Sci. Rep..

[17] Hason, M. *et al.* Bioluminescent zebrafish transplantation model for drug discovery. *Front. Pharmacol.* **13** (2022). 10.3389/fphar.2022.893655 PMC9086674 35559262

[18] Crouzier L (2021). Use of zebrafish models to boost research in rare genetic diseases. Int. J. Mol. Sci..

[19] Zhang Y (2017). ATP6V1H deficiency impairs bone development through activation of MMP9 and MMP13. PLoS Genet..

[20] Perles Z (2015). A human laterality disorder caused by a homozygous deleterious mutation in MMP21. J Med Genet.

[21] Guissart C (2018). Dual Molecular effects of dominant RORA mutations cause two variants of syndromic intellectual disability with either autism or cerebellar ataxia. Am. J. Hum. Genet..

[22] Bai H (2020). CRISPR/Cas9-mediated precise genome modification by a long ssDNA template in zebrafish. BMC Genomics.

[23] Rosello M (2021). Precise base editing for the in vivo study of developmental signaling and human pathologies in zebrafish. Elife.

[24] Rosello M (2022). Disease modeling by efficient genome editing using a near PAM-less base editor in vivo. Nat. Commun..

[25] Bradford YM (2022). Zebrafish information network, the knowledgebase for Danio rerio research. Genetics.

[26] Howe DG (2017). The zebrafish model organism database: New support for human disease models, mutation details, gene expression phenotypes and searching. Nucl. Acids Res..

[27] Hoshijima K (2019). Highly efficient CRISPR-Cas9-based methods for generating deletion mutations and F0 embryos that lack gene function in zebrafish. Dev. Cell.

[28] Feldwisch-Drentrup H (2022). Germany weighs whether culling excess lab animals is a crime. Science.

[29] Hose K, Nagel-Riedasch S, Schenkel J, Buch T (2022). Use surplus laboratory animals as animal feed. Lab. Anim..

[30] Abbott A (2020). Animal-research data show effects of EU’s tough regulations. Nature.

[31] Sofou K (2021). Bi-allelic VPS16 variants limit HOPS/CORVET levels and cause a mucopolysaccharidosis-like disease. EMBO Mol. Med..

[32] Kroll F (2021). A simple and effective F0 knockout method for rapid screening of behaviour and other complex phenotypes. Elife.

[33] Bek JW (2021). Lrp5 mutant and crispant zebrafish faithfully model human osteoporosis, establishing the zebrafish as a platform for CRISPR-based functional screening of osteoporosis candidate genes. J. Bone Miner. Res..

[34] Liu K, Petree C, Requena T, Varshney P, Varshney GK (2019). Expanding the CRISPR toolbox in zebrafish for studying development and disease. Front. Cell Dev. Biol..

[35] Kosuta C (2018). High-throughput DNA extraction and genotyping of 3dpf zebrafish larvae by fin clipping. J. Vis. Exp..

[36] Naganawa Y, Hirata H (2011). Developmental transition of touch response from slow muscle-mediated coilings to fast muscle-mediated burst swimming in zebrafish. Dev. Biol..

[37] Sztal TE, Ruparelia AA, Williams C, Bryson-Richardson RJ (2016). Using touch-evoked response and locomotion assays to assess muscle performance and function in zebrafish. J. Vis. Exp..

[38] LaFave MC, Varshney GK, Vemulapalli M, Mullikin JC, Burgess SM (2014). A defined zebrafish line for high-throughput genetics and genomics: NHGRI-1. Genetics.

[39] Asakawa K, Kawakami K (2008). Targeted gene expression by the Gal4-UAS system in zebrafish. Dev. Growth Differ..

[40] Okamoto H, Ishioka A (2010). Zebrafish research in Japan and the national bioresource project. Exp. Anim..

[41] Kay JN, Finger-Baier KC, Roeser T, Staub W, Baier H (2001). Retinal ganglion cell genesis requires lakritz, a zebrafish atonal homolog. Neuron.

[42] Auer TO (2015). Deletion of a kinesin I motor unmasks a mechanism of homeostatic branching control by neurotrophin-3. Elife.

[43] Westerfield, M. *The Zebrafish Book. A Guide for the Laboratory Use of Zebrafish (Danio rerio)* (2000).

[44] Koblan LW (2018). Improving cytidine and adenine base editors by expression optimization and ancestral reconstruction. Nat. Biotechnol..

[45] Don EK (2017). A Tol2 gateway-compatible toolbox for the study of the nervous system and neurodegenerative disease. Zebrafish.

[46] Aljiboury AA, Mujcic A, Cammerino T, Rathbun LI, Hehnly H (2021). Imaging the early zebrafish embryo centrosomes following injection of small-molecule inhibitors to understand spindle formation. STAR Protoc..

[47] Kluesner MG (2018). EditR: A method to quantify base editing from sanger sequencing. CRISPR J..

[48] Schindelin J (2012). Fiji: An open-source platform for biological-image analysis. Nat. Methods.

[49] Lambert CJ (2018). An automated system for rapid cellular extraction from live zebrafish embryos and larvae: Development and application to genotyping. PLoS ONE.

[50] Breacker C, Barber I, Norton WHJ, McDearmid JR, Tilley CA (2017). A low-cost method of skin swabbing for the collection of DNA samples from small laboratory fish. Zebrafish.

[51] van Eeden FJ (1996). Genetic analysis of fin formation in the zebrafish, Danio rerio. Development.

[52] Asakawa K, Kawakami K (2009). The Tol2-mediated Gal4-UAS method for gene and enhancer trapping in zebrafish. Methods.

[53] Prospéri M-T, Pernier J, Lachuer H, Coudrier E (2021). Plekhh, a partner of myosin 1 and an effector of EphB2, controls the cortical actin network during cell repulsion. J. Cell Sci..

[54] Hu JH (2018). Evolved Cas9 variants with broad PAM compatibility and high DNA specificity. Nature.

[55] Walton RT, Christie KA, Whittaker MN, Kleinstiver BP (2020). Unconstrained genome targeting with near-PAMless engineered CRISPR-cas9 variants. Science.

[56] Komor AC, Kim YB, Packer MS, Zuris JA, Liu DR (2016). Programmable editing of a target base in genomic DNA without double-stranded DNA cleavage. Nature.

[57] Beedgen L (2022). A rapid and simple procedure for the isolation and cultivation of fibroblast-like cells from medaka and zebrafish embryos and fin clip biopsies. Lab. Anim..

[58] Lancaster MA, Knoblich JA (2014). Generation of cerebral organoids from human pluripotent stem cells. Nat. Protoc..

[59] Zhang X, Zhang Z, Zhao Q, Lou X (2020). Rapid and efficient live zebrafish embryo genotyping. Zebrafish.

[60] Sneddon LU, Halsey LG, Bury NR (2017). Considering aspects of the 3Rs principles within experimental animal biology. J. Exp. Biol..

[61] Veldman MB, Lin S (2008). Zebrafish as a developmental model organism for pediatric research. Pediatr. Res..

