# Supplementary material for: A minimally invasive fin scratching protocol for fast genotyping and early selection of zebrafish embryos
Source: Sci Rep. 2022 Dec 30;12:22597. doi: 10.1038/s41598-022-26822-7 (PMC9803660; doi:10.1038/s41598-022-26822-7)
Supplement: Supplementary file 2 — Supplementary Information 2. [file 41598_2022_26822_MOESM2_ESM.pdf]

Supplementary Material

-Supplementary Figure 1. Details of the tail fin scratching (FS) procedure in zebrafish embryos and tissue material collection for genome extraction.

-Supplementary Figure 2. Results from a single round of PCR of endogenous target gene fragments of different sizes from the genomic DNA obtained by applying the FS procedure on 48 hpf zebrafish embryos.

-Supplementary Figure 3. Results from one and two rounds of PCR of the transgenic Gal4 fragment and an endogenous gene fragment of approximately 1000 bps from the genomic DNA obtained by applying the FS procedure on 48 hpf zebrafish embryos.

-Supplementary Figure 4. Results from one and two rounds of PCR of an endogenous target gene fragment of 200 bps from the genomic DNA obtained by applying the FS procedure on 48 hpf zebrafish embryos.

-Supplementary Figure 5. Screening of *Lak*$^{+/-}$ 48hpf fish by PCR followed by enzymatic digestion using the gDNA derived from the FS protocol.

-Supplementary Figure 6. Examples of genotyping results obtained by sequencing the *tyr* target locus from FS-derived genomic DNA of 48hpf crispant fish showing mild or severe pigmentation phenotype.

-Supplementary Table 1. List of the primers used in this study to amplify transgene and mutant alleles.

-Source data (uncropped gel images of Supplementary Figures)

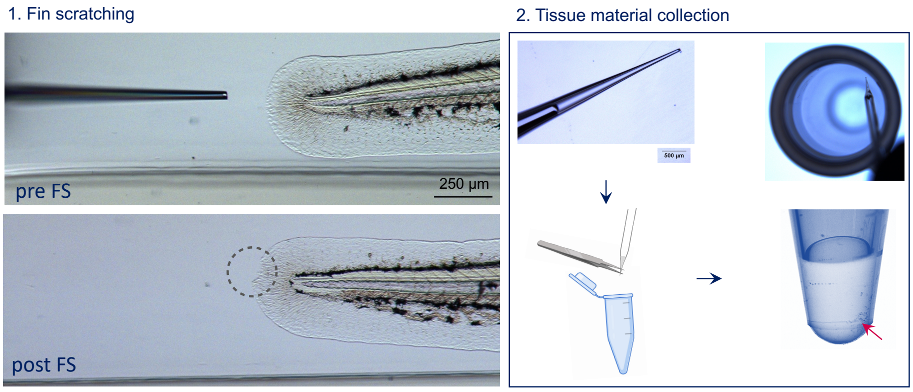

Supplementary Figure 1. Details of the tail fin scratching (FS) procedure in zebrafish embryos and tissue material collection for genome extraction. On the left (1), representative bight field images of a single embryo's tail before (up, "pre") and after (below, "post") FS obtained with the pooled capillary shown in the image. Scratched tissue can be observed (dashed circle). On the right (2), a close-up of the capillary after FS is shown. Liquid collected during scratching by capillarity within the needle is observed. The schematic illustration shows the retrieval of the FS-derived material by cutting the tip of the capillary and releasing it into a standard collection tube. After the procedure is complete the presence of glass from the capillary can be observed at the bottom of the collection tube (dark pink arrow).

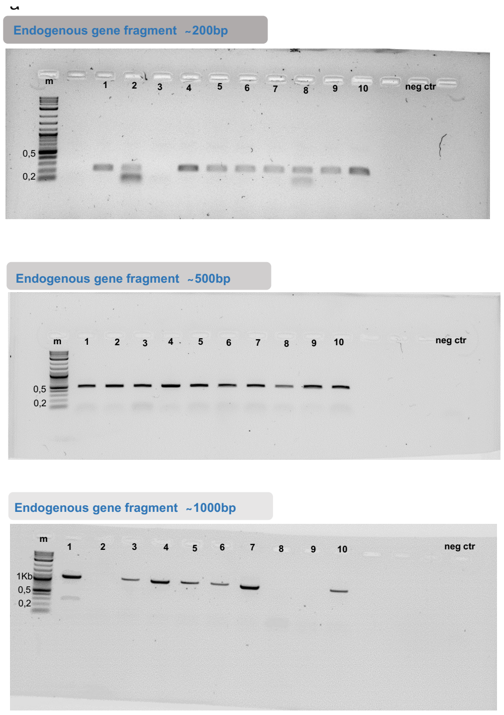

b

Size of the endogenous gene fragments	Number of successful fragment amplification / Number of total samples analyzed	Efficiency %
203 bp	29/30	97%
527 bp	30/30	100%
1113 bp	19/30	63%

Supplementary Figure 2. Results from a single round of PCR of endogenous target gene fragments of different sizes from the genomic DNA obtained by applying the FS procedure on 48 hpf zebrafish embryos. (**a**) Amplification of gene fragments of approx. 200, 500 and 1000 bps. (**b**)

Table showing the occurrence of successful amplifications on the total number of embryos subjected to FS per each target. The value in percentage indicating the efficiency is shown on the last column. In all the images the numbers correspond to different individuals (embryos at 48hpf). N = 30 individuals in total. The gels are representative results of two independent experiments. Replicates contributing to the percentage in the table are shown in Supplementary Figure 3a (lower gel for 200 bps), Source Data file (for 500 bps) and Supplementary Figure 3c (for 1000 bps). "M" indicates the DNA ladder, and the 0.2, 0.5 and 1 kb bands are shown.

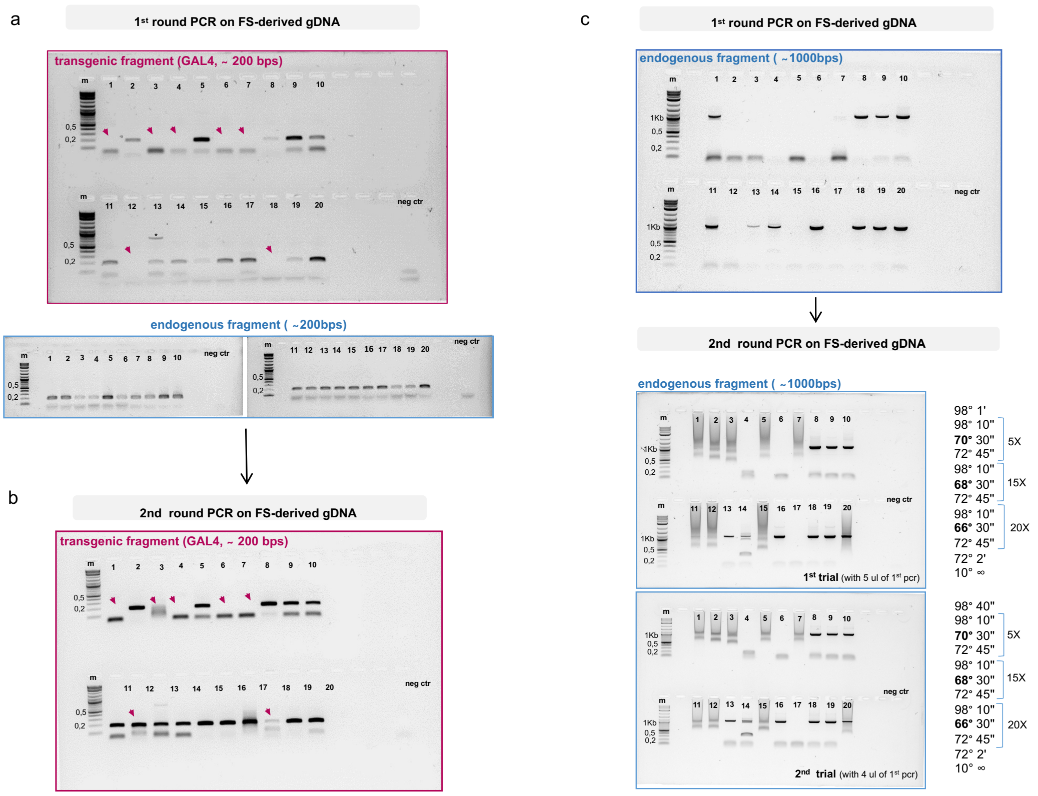

Supplementary Figure 3. Results from one and two rounds of PCR of the transgenic Gal4 fragment and an endogenous gene fragment of approximately 1000 bps from the genomic DNA obtained by applying the FS procedure on 48 hpf zebrafish embryos. (**a**) Gal4 target amplification from GFP negative *SAGFF73A:Gal4 x UAS:Gtuba* incross fish after one round of PCR. The lower panels show the successful amplification of endogenous targets of approx. 200 bps obtained from the FS-derived gDNA of the same fish analyzed for Gal 4. (**b**) Results of the "PCR on PCR" (2nd round of PCR) on Gal4 from the PCR samples shown in a. Pink arrows indicate negative results in the first round of PCR. (**c**) Target amplification of an endogenous gene fragment of approx. 1000 bps after one (up) or two (down) rounds of PCR on the gDNA extracted by FS protocol from NHGRI fish. For the 2nd round of PCR the results and settings of two different PCR trials are shown. For all the gels reported here, the n of embryos screened = 20. In all the images the numbers correspond to different individuals (embryos at 48hpf) and the 2nd round of PCR is performed using the target amplicons from the 1st round of PCR as starting material. The gel is representative of one (upper gel in a, b and lower panel in c) or two

(upper panel in c) independent experiments. “M” indicates the DNA ladder, and the 0.2, 0.5 and 1 kb bands are shown.

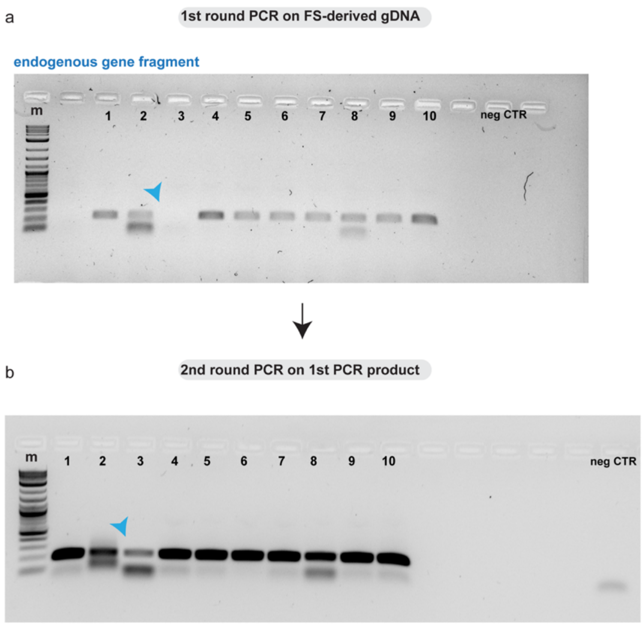

Supplementary Figure 4. Results from one and two rounds of PCR of an endogenous target gene fragment of 200 bps from the genomic DNA obtained by applying the FS procedure on 48 hpf zebrafish embryos. Endogenous gene target amplification after one (**a**) or two (**b**) rounds of PCR on the gDNA extracted by FS protocol in NHGRI fish. The numbers correspond to different individuals (embryos at 48hpf). The 2nd round of PCR is performed using the target amplicons from the 1st round of PCR as starting material. Amplification is evident for individual 3 after the second round of PCR (compare blue arrow between a and b). Note that a is the same gel shown in Figure S2a, shown here to highlight the failure of target amplification in one individual which was rescued via re-amplification. The upper gel is a representative result of two independent experiments (replicate in Supplementary Figure 2a), the “2nd round of PCR (lower gel) is a single experiment. M” indicates the DNA ladder.

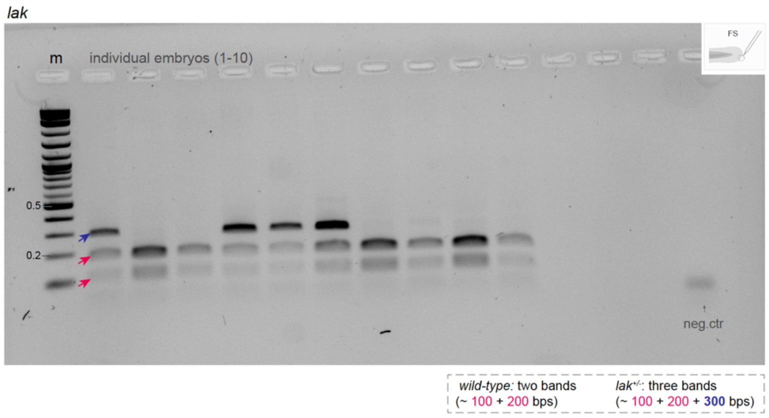

Supplementary Figure 5. Screening of *Lak*$^{+/-}$ 48hpf fish by PCR followed by enzymatic digestion using the gDNA derived from the FS protocol. Single genotyping results of 48 hpf fish (n=10)

screened for the presence of the heterozygous allele *lak +/-* following Kay et al., 2001 and Auer et al., 2015. The distinctive presence of WT and mutant bands is indicated by dark pink and blue arrows, respectively. "M" indicates the DNA ladder, and the 0.2 and 0.5 kb bands are shown.

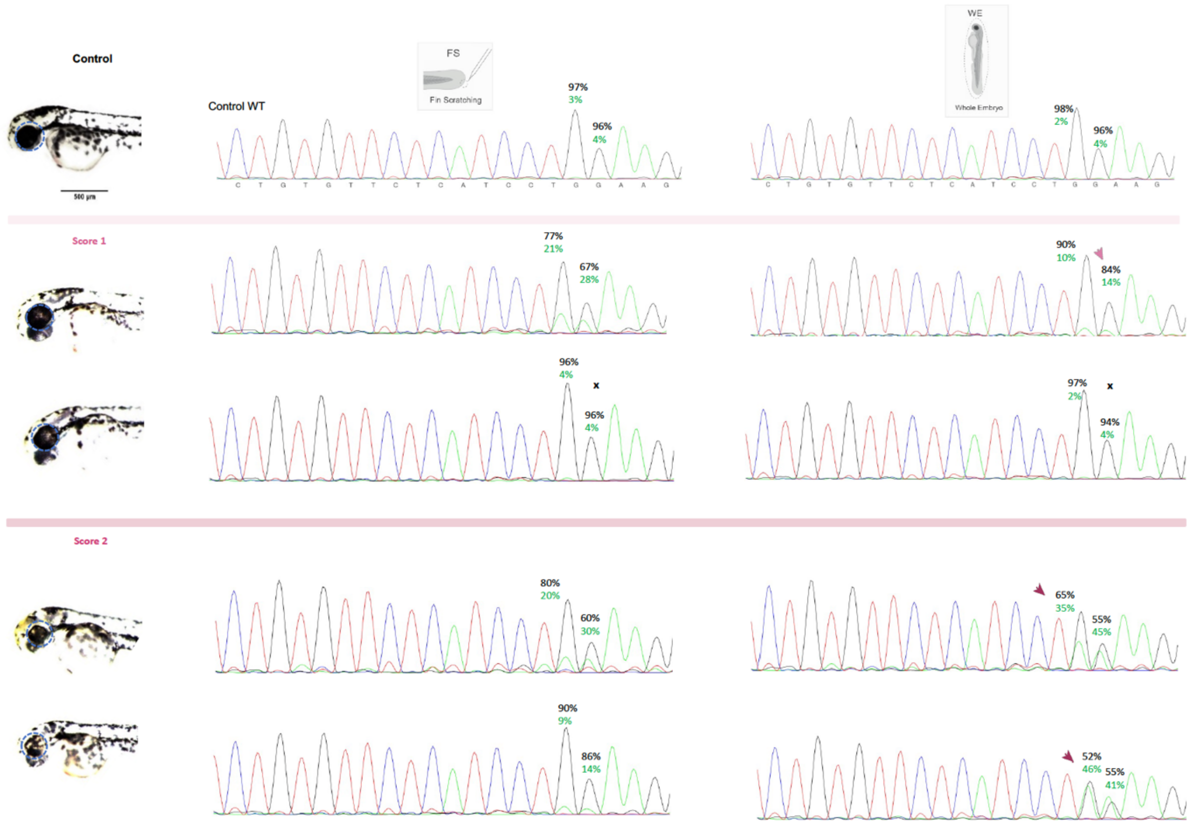

Supplementary Figure 6. Examples of genotyping results obtained by sequencing the *tyr* target locus from FS-derived genomic DNA of 48hpf crispant fish showing mild or severe pigmentation phenotype. Additional examples of the genotyping-phenotyping performance ability in Be-CRISPR/Cas9 G0 fish by using FS- compared to WE-based gDNA preparation from the same individuals. Representative images and chromatograms are shown of fish exhibiting mild (score 1) or more severe (score 2) phenotypes and for which genotyping was first performed by performing Sanger sequencing on PCR bands obtained via FS (left) and then by WE (right). Refer to Figure 2 for other examples and overall data quantification. Dark pink arrows indicate high rate of G>A (C>T) conversion of the targeted locus which leads to a premature STOP codon in the *tyrosinase (tyr)* coding sequence. Percentage of base conversion and of residual G were calculated by EditR and are shown in green and black, respectively. "x" indicates normal chromatogram (no appreciable base conversion). A reference negative control fish and sequence from a not-injected individual is shown in the upper panel.

GFP Fw	5'- CGTAGCCTTCGGGCATGGCGGAC-3'
GFP Rv	5'- CTGGTCGAGCTGGACGGCGACG-3'
Gal4 Fw	5'-GACATTTGCAGACTGAAGAAACTGA-3'
Gal4 Rv	5'-TCATATCCAGGTCCTCTCTAGGAAA-3'
Tyr Fw	5'-AACCACCATAAGCCCCGATTATATG-3'
Tyr Rv	5'-CCGCCCCTAGAACTAACATTCTTG-3
Plekhh1 Fw	5'-GACAGAGACCACTCCTCTGATGAA-3'
Plekhh1 Rv	5'- CTTCTGTAATCTCAGACCCCTCCA-3'
Lak Fw	5'-CCGGAATTACATCCCAAGAAC-3'
Lak Rv	5'-GGCCATGATGTAGCTCAGAG-3'

Table S1: list of the primers used in this study to amplify transgene and mutant alleles.

Source data (uncropped gels relative to Supplementary Figure 4)

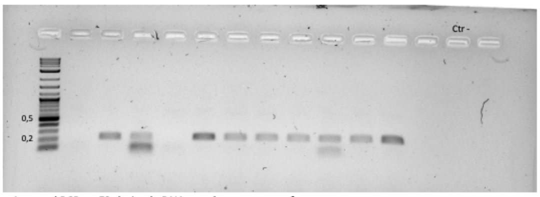

1s round PCR on FS-derived gDNA _ endogenous gene fragment

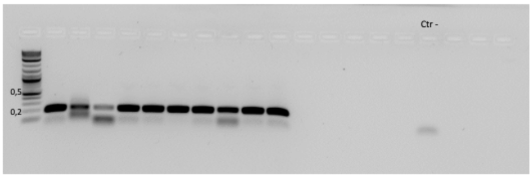

2nd round PCR on 1st PCR product_ endogenous gene fragment

Source data relative to Supplementary Figure 4. Uncropped image of the agarose gel showing the 1st and 2nd round of PCR on the endogenous gene fragment of approx. 200 bps in different zebrafish embryos whose genomic DNA was derived by FS.
